# Exploring older adults’ lived experiences of COVID-19: A narrative inquiry study

**DOI:** 10.1093/geroni/igab046.3210

**Published:** 2021-12-17

**Authors:** Tia Rogers-Jarrell, Brad Meisner

**Affiliations:** 1 York University, Hamilton, Ontario, Canada; 2 York University, Toronto, Ontario, Canada

## Abstract

COVID-19 dramatically changed daily life for older adults in numerous and complex ways. Research is calling for an understanding on how COVID-19 has and will impact aging, and older adults’ lived experiences with aging, within the context of the pandemic. Social and physical distancing guidelines have put older adults at an increased risk for social isolation. Intergenerational tensions have also intensified during the pandemic, and early research states the labeling of older adults as a homogenous and “vulnerable” group can lead to an increased risk of ageism in their communities. Therefore, the purpose of this study is to explore how community-dwelling older adults (ages 65 and greater) experience daily life amid the COVID-19 pandemic using a biopsychosocial approach. This study employs a critical qualitative narrative inquiry design. Data will be collected through solicited diaries and semi-structured individual interviews (via telephone and video conferencing software). Data will be analyzed thematically and involve a re-storying of the findings. Preliminary results will be presented and discussed. This study aims to inform new and critical perspectives that broaden our understanding of how the overall health, wellness, and quality of life of older adults can be supported. Findings contribute to the current and developing knowledge of older adults’ first-person accounts of their experiences within the COVID-19 pandemic.

